# Host genetics of Epstein–Barr virus infection, latency and disease

**DOI:** 10.1002/rmv.1816

**Published:** 2014-11-27

**Authors:** Charlotte J Houldcroft, Paul Kellam

**Affiliations:** 1 Wellcome Trust Sanger Institute, Wellcome Trust Genome CampusCambridge, UK; 2 Division of Biological Anthropology, Department of Archaeology and Anthropology, University of CambridgeCambridge, UK; 3 Department of Infection, Division of Infection and Immunity, University College LondonLondon, UK

## Abstract

Epstein–Barr virus (EBV) infects 95% of the adult population and is the cause of infectious mononucleosis. It is also associated with 1% of cancers worldwide, such as nasopharyngeal carcinoma, Hodgkin's lymphoma and Burkitt's lymphoma. Human and cancer genetic studies are now major forces determining gene variants associated with many cancers, including nasopharyngeal carcinoma and Hodgkin's lymphoma. Host genetics is also important in infectious disease; however, there have been no large-scale efforts towards understanding the contribution that human genetic variation plays in primary EBV infection and latency. This review covers 25 years of studies into host genetic susceptibility to EBV infection and disease, from candidate gene studies, to the first genome-wide association study of EBV antibody response, and an EBV-status stratified genome-wide association study of Hodgkin's lymphoma. Although many genes are implicated in EBV-related disease, studies are often small, not replicated or followed up in a different disease. Larger, appropriately powered genomic studies to understand the host response to EBV will be needed to move our understanding of the biology of EBV infection beyond the handful of genes currently identified. Fifty years since the discovery of EBV and its identification as a human oncogenic virus, a glimpse of the future is shown by the first whole-genome and whole-exome studies, revealing new human genes at the heart of the host–EBV interaction. © 2014 The Authors. *Reviews in Medical Virology* published by John Wiley & Sons Ltd.

Epstein–Barr (EBV) is a human gamma herpesvirus, infecting over 95% of adults by the age of 30 years [1,2]. Initial infection with EBV typically occurs in childhood, transmitted through saliva [3]. Childhood infections are usually clinically silent or difficult to distinguish from other mild viral infections [4,5]. Worldwide, EBV is estimated to cause 1% of cancers, the fourth most common infectious cause of cancer in 2002 [6].

EBV's primary tropism is for B and epithelial cells but can also infect NK, T and smooth muscle cells, with the ability to cause oncogenesis in all of these cell types [7]. Following primary infection, EBV establishes a latent infection and persists within memory B cells at low levels, allowing a lifelong infection to be established that the immune system cannot clear. EBV's biphasic life cycle has two stages: the lytic cycle allows EBV to productively infect new cells and new hosts, whereas the latency cycle is vital to allow persistence of the virus within infected cells. Latency can be further divided into four types (Table 1) with restricted viral gene expression to avoid immune surveillance. Viral gene expression changes when EBV enters the lytic cycle [8,9].

**Table 1:** Latency profiles of EBV-infected cells

Latency type	Features*	Gene expression profile
Type 0	Naturally EBV-infected B cells within some healthy individuals [107]	EBERs (LMP2A)
Type I	Memory B cells, EBV + BL and BL-derived cell lines [108]	EBERs, EBNA1
Type II	EBV + NPC, EBV + GC, HL and T-cell lymphoma	EBERs, EBNA1, LMP2A, LMP1
Type III	LCLs; B-cell tumours in immunosuppressed individuals, for example, PTLD, AIDS-related immunoblastic lymphomas	EBERs, EBNAs, LMPs

* Adapted from Kutok and Wang [109] and Young *et al.* [110].

## Understanding the Host Genetics of Susceptibility to Infectious Disease

Host genetic variation is well established as a component of infectious disease pathogenesis and has the potential to lead to new tests and treatments for primary infections and their long-term complications, and this is no less true of EBV [10]. Candidate gene studies have highlighted a number of common, high-penetrance human genetic variants associated with infection and disease resistance. A deletion in chemokine (C-C Motif) receptor 5 (*CCR5*) gives resistance to certain strains of HIV [11,12], whereas a deletion in fucosyltransferase 2 (*FUT2*) conveys resistance to symptomatic norovirus infection. Rare single gene mutations, such as primary deficiencies of the TLR signalling system, specifically the *TLR3–Unc93b–TRIF–TRAF3* pathway, have been associated with increased susceptibility specifically to herpesvirus infection, particularly herpes simplex virus encephalitis [13].

GWAS are being used to understand the host genetics of virus infection. They require no prior knowledge of which regions of the genome are implicated in disease aetiology; dense genotyping studies allow association results to be localised to smaller regions of the genome; and they are a cost-effective way of interrogating the genetic contribution to a trait of interest [14]. Host genetic susceptibilities to hepatitis B, hepatitis C and HIV have all been highlighted by GWAS. These studies have been successful even in the face of pathogen genome variation, a potential confounding factor.

Whole-exome or genome sequencing is another powerful approach to identifying rare, often large effect size or high-penetrance mutations within largely coding readings of the human genome and has been used in a clinical setting to identify the aetiology of rare genetic diseases [15]. For individual cases, sequencing can identify atypical presentations of genetic disorders with EBV involvement such as XLP [16] or previously unknown mutations that mimic known EBV-susceptibility conditions [17], allowing the correct diagnosis and treatment. Since mutations in the *PRF1* gene that reduce or knock out *PRF1* function have been identified as a cause of some EBV-driven lymphoproliferations, autologous T-cell gene therapy that restores *PRF1* expression is being used to treat these patients [18]. At a population level, understanding the genetic basis of susceptibility to EBV infection or EBV-related disease will help us to pinpoint those who would benefit most from any future vaccine for EBV [19] and may contribute to the development of new treatment strategies.

## The Host Genetics of Primary Epstein–Barr Virus Infection, Latency and Complications of Primary Infection

### Epstein–Barr virus antibody response

EBV antibody responses are epidemiologically linked to the development of BL [20] and HL [21]. Studying the host genetics of EBV antibody response is seen as an intermediate step to identifying the genetics of EBV-related disease development.

A number of human genetic variants have been associated with increased anti-VCA-IgA levels in a candidate gene study of polymorphisms in the HRRS, namely, *MDC1*, *RAD54L*, *TP53BP1*, *RPA1*, *LIG3* and *RFC1* [22]. EBV utilises the HRRS during lytic reactivation [23], and increased lytic reactivation may be inferred to increase anti-VCA-IgG levels. Anti-VCA-IgG antibody levels are also correlated with variants within *IL10* [24], as well as human leukocyte antigen (HLA)-DR2 in healthy blood donors [25]. SNP rs16944 in *IL1B* is associated with EBV seropositivity in healthy blood donors [26]. Genetic variants in the exons of *MBL2* that lead to mannose-binding lectin deficiency have been associated with lower anti-VCA-IgG antibody titres [27]. Genome-wide analysis of anti-EBNA-1 antibody levels in more than 1300 Mexican Americans identified polymorphisms in genes within the HLA and associated regions of chromosome 6 that were correlated with anti-EBNA-1 levels, namely, proline-rich coiled-coil 2A, *EHMT2*, butyrophilin-like 2 (MHC class II associated) (*BTNL2*), HLA-DRA, HLA-DRB9 and HLA-DRB1, associations that were replicated in a larger cohort. Using 60 large family pedigrees, anti-EBNA-1 levels were shown to be 43% heritable, and discrete anti-EBNA-1 status (seropositive vs seroindeterminate vs seronegative) to be 68% heritable [28]. A study of EBV-antibody-negative blood donors aged over 60 years (with no detectable EBV DNA in blood) compared the frequencies of HLA alleles HLA-C −35 (associated with increased T-cell signalling) and HLA-Bw4 between negative cases and age-matched EBV-positive controls. The HLA-C −35 allele was more frequent in EBV-positive donors, whereas HLA-Bw4 was more frequent in EBV-negative blood donors. However, because of the difficulties in finding EBV-negative study participants in this age group, only a small number of cases (17) were included [29].

From these studies, it is clear that variation in EBV serostatus has a host genetic component, most reliably identified by large studies. It is less clear whether the host genetic variants underlying control of EBV antibody response also contribute to the development of those lymphomas epidemiologically linked to high EBV antibody levels.

### Infectious mononucleosis and complications of primary Epstein–Barr virus infection of B cells

IM is usually a benign, self-limiting lymphoproliferative disease most common in the Western world, which occurs in between 25% and 70% of young adults following primary EBV infection [30]. Symptoms of IM appear around a month after primary infection and range from benign (lymphadenopathy, sore throat, fever and fatigue) to severe (fulminant hepatitis, liver necrosis and/or HLH (see following)). The appearance and increase of EBV-specific T lymphocytes leads to a gradual decline in symptoms [31]. Rarely, severe or fatal IM develops if no successful EBV-CTL (cytotoxic T lymphocyte) response is mounted, which may be sporadic or linked to genetic disorders such as XLP (see following) [31]. Certain cancers and other chronic conditions are epidemiologically linked to IM [32], with independent epidemiological links between HL and MS following a clinical diagnosis of IM.

Two large population studies of IM concordance in twins [33] and first-degree relatives [34] have suggested a heritable genetic component to IM, and candidate gene studies have identified IM-associated regions of the human genome. HLA class I polymorphisms initially identified in EBV-positive HL (see following) are associated with an increased risk of developing IM [35]. Individuals homozygous for allele 1 of the STR D6S510 or allele 3 of STR D6S265 had an OR of 2.7 for IM development. SNPs rs253088 on chromosome 5 and rs6457110 on chromosome 6 were also associated with IM development [5]. SNP rs1982073 in *TGFB1* were found at higher frequencies in IM cases than in healthy EBV-positive controls [36]. Finally, the ATA haplotype of *IL10* promoter SNPs rs1800896, rs1800871 and rs1800872 has been associated with risk of IM [37].

Overall, relatively little is known about the common genetic basis of susceptibility to primary EBV infection, despite the genetic contribution suggested by twin and family studies of IM concordance. There has been greater success in identifying rare mutations associated with severe responses to primary EBV infection of B cells, of which XLP is perhaps the best known. XLP is a genetic disorder with a prevalence of approximately 1 in 1 million men [38]. People with XLP are clinically healthy in 90% of cases until primary infection with EBV, which results in fulminant IM [39]. Mutations in two human genes have so far been implicated in XLP: *SH2D1A* and *XIAP* [40]. Some affected individuals do not carry these mutations, suggesting further genetic factors are involved in this disease.

Exome and genome sequencing have identified four further disorders involving genetic susceptibility to EBV infection: XMEN, *ITK* deficiency, *CORO1A* deficiency and *PRKCD* deficiency. XMEN is caused by an X-linked mutation identified by exome sequencing. Mutations within *MAGT1* impair magnesium transporter function, necessary for T-cell antigen receptor stimulation, leading to poor T-cell function. Whereas this T-cell deficiency leads to a general immunodeficiency in affected patients, chronic EBV infection of B cells is a particular characteristic [41]. *ITK* deficiency, caused by autosomal recessive mutations in the *ITK* gene, leads to high EBV DNA loads in peripheral blood and B cells and treatment-resistant, fatal B-cell proliferation following initial EBV infection. It has been detected in five families [17,42,43]. *CORO1A* deficiency, described as ‘leaky SCID’ (severe combined immune deficiency), is linked to both primary immunodeficiency and EBV-associated B-cell proliferations. A homozygous missense mutation in *CORO1A* led to a less stable form of the coronin protein, causing a T-cell deficiency [44]. Of note is a small gene expression study that found that *CORO1A* was over-expressed in LCLs that were non-permissive for EBV lytic reactivation and had low EBV genome loads per cell. This suggests a role for coronin in EBV control in B cells, as well as in T-cell development [45]. A homozygous mutation located in *PRKCD* causes an autoimmune lymphoproliferative syndrome, with persistent high EBV DNAemia, low PRKCD protein expression and reduced NK cell cytolysis. This suggests that *PRKCD* plays an important role in controlling infected B-cell proliferation and EBV load following primary infection [46]. Sequencing of rare, extreme reactions to primary EBV infection is enhancing our understanding of EBV control in immunocompetent individuals.

### Chronic Epstein–Barr virus infection of T and NK cells

EBV is primarily B lymphotropic, but in the context of immune suppression or genetic susceptibility, infection of T and NK cells may occur. One such condition is CAEBV. When primary EBV infection symptoms do not spontaneously resolve in otherwise immunocompetent individuals, with infection of T and NK cells, recurrent or chronic bouts of IM-like illness result in abnormal patterns of EBV antibodies. CAEBV patients have very high blood EBV loads [31]. CAEBV is more prevalent in south Asia, and particularly Japan, suggesting a particular host genetic background common in this region may also be a risk factor for CAEBV development [47]. Mutations in the *PRF1* gene were found in one patient who died following the onset of CAEBV [48], and this disorder is increasingly treated by HCST [49].

*PRF1* mutations are also a feature of HLH, a rare autosomal recessive disorder affecting between 0.12 per 100,000 children in Sweden and 0.342 per 100,000 children in Japan. Macrophages and CTL are hyperactivated in HLH, leading to an inflammatory response extreme enough to cause multiple organ failure. Primary HLH is typically diagnosed in early childhood but can develop later in life as secondary HLH, which may be triggered by infection. EBV is the most common viral trigger of primary and secondary HLH [50], infecting T and NK cells leading to clonal lymphoproliferation [51]. Genetic studies are blurring the distinctions between primary and secondary HLH [52], as a number of previously healthy adults (some aged over 50 [53]) initially diagnosed with secondary HLH have been found to carry mutations in the same genes that cause primary HLH in infants.

Mutations in *PRF1* have been found in adults with reactivated EBV infections and HLH [54], with significant genetic overlap between HLH and CAEBV. *PRF1* interacts with GZMB in cytotoxic cell death, and plasma granzyme expression is elevated in herpesvirus infection [55], so the link between *GZMB* polymorphisms and EBV-positive HLH has been investigated. In a small study where 20 HLH cases were compared with IM cases and healthy controls, the wildtype ‘QPY’ (rs8192917, rs11539752 and rs2236338) haplotype of *GZMB* was statistically significantly over-represented in HLH cases compared with the ‘RAH’ triple-mutated haplotype that has no apoptotic function [56]. A study of killer cell immunoglobulin-like receptor (KIR) alleles also found KIR2DS5 to be significantly enriched in people with HLH compared with children with IM or healthy age-matched controls [57]. EBV-associated HLH is also linked to polymorphisms in *STX11* [58], *UNC13D* [59] and *RAB27A* [60]. A common thread is the failure of cellular cytotoxicity, particularly granule-mediated pathways, underlying the genetic basis of HLH. This indicates that further genes with cellular cytotoxic functions may be important to the control of EBV infection and the pathogenesis of HLH.

In summary, failures in T-cell activity or activation are a feature of many diseases that have chronic active EBV infection as a major symptom, suggesting that genes and pathways involved in T-cell development and regulation may feature further mutations that influence the outcome of primary EBV infection. Further investigation of primary immunodeficiencies is likely to uncover more host genes associated with EBV infection.

## The Host Genetics of Epstein–Barr Virus-Related Lymphoproliferations and Cancers

### Post-transplant lymphoproliferative disorders

PTLDs occur most commonly after EBV seronegative hosts receive EBV seropositive donor organs (10% of recipients [61]) or during the profound immune suppression following HCST (1% of cases [62]) [63]. The majority of PTLDs are of B-cell origin (85%), of which 80% are EBV-positive, but may also involve other cell types such as T cells (10–15%, of which 30% are EBV-positive) [61,64]. There are a range of lymphomas within the category of B-cell origin PTLD, and the broad grouping of these sequelae may confound efforts to identify the host genetic risk factors involved.

Candidate gene studies of transplant recipients show HLA haplotypes, such as HLA-A26 and B38, that are associated with a higher risk of developing EBV-positive B-cell origin PTLD [65]. Following transplantation, some individuals develop chronic high EBV loads and progress to PTLD, whereas others resolve their high virus loads relatively quickly. A sudden sharp rise in EBV load is predictive of the development of EBV-positive PTLD. The HLA-A02 allele was found in 80% of individuals with chronic high viral loads, whereas 71% of individuals who resolved their high viral load carried allele HLA-B08 [66]. Other small studies have identified HLA-DR7 as a risk factor for the development of a symptomatic EBV infection in transplant recipients [67]. *IL1RN* has a 86 bp VNTR in the second intron which is associated with EBV viraemia in liver transplant recipients, as is *IL1B* SNP rs16944 [68]. *TGFB1* and *IL10* polymorphisms were associated with developing EBV-positive PTLD in a candidate gene study of 38 late-onset PTLD cases and 400 PTLD-free solid organ transplant recipients [69]. In contrast, in a second smaller study, PTLD cases and PTLD-free transplant recipient controls did not find a link between lymphoma occurrence or survival and polymorphisms in *IL10* and *TGFB1*, but in this study PTLD cases were not divided by EBV status [70]. Variants within tumour necrosis factor alpha (*TNF*) and tumour necrosis factor receptor I (*TNFRSF1A*) have been associated with EBV-positive PTLD. Increased serum levels of TNF were found in patients with PTLD versus EBV-positive PTLD-free solid organ recipients [71]. An *IFNG* low producer genotype [72] was found more often in PTLD cases following paediatric liver transplant (67%) than in PTLD-free liver recipients (33%), although the sample sizes for both groups were small, namely 6 and 53 patients, respectively. Although variants in the HLA regions and in a variety of regulatory and inflammatory cytokine genes have been implicated in PTLD pathogenesis, there is as yet no clear picture of the host genetics underlying PTLD risk or whether the studies presented earlier represent causative variants or false-positive associations.

### Epstein–Barr virus-positive Hodgkin's lymphoma

HL is a tumour originating from B lymphocytes. Not all HLs are EBV-positive, and the percentage of lymphomas positive for the virus varies by HL subtype, with EBV found in 50–80% of mixed-cellularity type HL [31]. The EBV-positive HL is more common than EBV-negative HL in young children and older adults, with the opposite pattern in adolescents [32]. EBV is also epidemiologically linked with HL, with a relative risk of HL following IM of 2.55 [73].

HLA polymorphisms associated with the genetic risk of developing IM are also associated with developing EBV-positive HL [35,74]. A large GWAS of EBV-positive and EBV-negative HL identified variants rs2734986 in HLA-A and rs6904029 in HLA complex group 9 (*HCG9*) as associated with EBV-positive HL [75]. The rs1800896 *IL10* promoter SNP, associated with high *IL10* expression, is more common in EBV-positive HL than EBV-negative cases [76]. The CC allele of *IL6* SNP rs1800795 was associated with a lower risk of developing EBV-positive HL (OR = 0.29) as a young adult, on the basis of a study of monozygotic and dizygotic twins and matched controls [77]. A small candidate gene study of *FCGR2A* (known as CD32) examined the frequency of SNP rs1801274 in classical HL [78]. This polymorphism leads to a histidine to arginine (H to R) substitution within *FCGR2A* that decreases the affinity of the receptor for human immunoglobulin G. When 23 EBV-positive and 81 EBV-negative HL cases were compared, 87% of EBV-positive cases carried at least one arginine-encoding allele compared with 68% of EBV-negative HL (p = 0.06); this gene has not been associated with HL by GWAS, although larger candidate genes studies have associated it with lymphoma risk [79]. GWAS reinforce the role of HLA polymorphisms in the development of HL; further large-scale genetic studies will be required to disentangle the contributions of cytokine polymorphisms to HL risk.

### Low-grade B-cell lymphoma

There are many B-cell lymphomas, not classically associated with EBV, in which EBV may play a pathogenic role, including chronic lymphocytic leukaemia, splenic marginal zone lymphoma, mantle cell lymphoma, hairy cell leukaemia and follicular lymphoma. In a small study of patients with various low-grade B-cell lymphomas, two polymorphisms in *FCGR2A* (previously studied in HL [78]) were found at different frequencies between EBV-positive and EBV-negative low-grade B-cell lymphoma. Allele R113 was more common in EBV-positive patients (84.2%) than in EBV-negative patients (28.5%). The R113 polymorphism also correlated with expression of EBV latent gene LMP1 in this study [80].

### Nasopharyngeal carcinoma

NPC occurs in the epithelium of the nose and throat and can be grouped as two broad subtypes—keratinising (differentiated epithelial cells) and non-keratinising (poorly or undifferentiated epithelial cells). Of non-keratinising NPC cases, 90% to 100% are EBV-associated, and between 30% and 100% of keratinising cases are EBV-associated [8]. The high prevalence of NPC in East Asia, and Central and South America, may be due to local host susceptibility genotypes that are absent or less common in other populations [81]. NPC has a number of non-viral risk factors that may partly explain its varied geographic incidence rate, but recent genetic studies suggest NPC arises in part from an interaction between the virus and host genotype [82]. A functional polymorphism in *MAP2K4*, a candidate tumour suppressor gene, was found at different frequencies in 1200 NPC patients and 1300 controls. The G variant of rs3826392 is protective against NPC (OR = 0.78), particularly in EBV-negative individuals (OR = 0.51), but does not provide statistically significant protection against NPC in EBV-positive individuals (OR = 1.05) [83]. There is as yet no functional evidence of a link between *MAP2K4* and protection against EBV infection or NPC development, only reports of increased *MAP2K4* expression in EBV-positive T and NK lymphoma-derived cell lines [84]. In a small candidate gene study of Portuguese patients with NPC, homozygosity at allele A2 (two VNTR copies) of *IL1RN* was strongly associated with the non-keratinising form of NPC (OR = 4.08) when the allele frequency was compared with healthy controls [85]; the same polymorphism was associated, but not statistically significantly, with EBV seropositivity in a small sample of blood donors, highlighting the problem of investigating candidate gene associations in small studies [26]. A T/C polymorphism within the 5′ untranslated region of complement component receptor 2 (*CR2*) showed an association with NPC in a case–control analysis of candidate polymorphisms in a Cantonese population. In this study of approximately 500 cases and 400 controls, the C allele of SNP rs3813946 was associated with sporadic (non-familial) NPC (OR = 1.43) [86]. Following up a number of SNPs within the HLA region identified by three previous NPC GWAS, Tang and colleagues compared allele frequencies between 1400 EBV IgA-VCA-positive NPC cases, 1300 EBV-positive controls and 1300 EBV-negative controls (defined by the presence of anti-VCA-IgA in blood). They found that alleles HLA-A02:07 and 33:03 were at statistically significantly different frequencies when NPC cases were compared with EBV-negative controls but did not significantly differ between NPC cases and EBV-positive controls. In the HLA-B locus, alleles 27:04, 46:01 and 58:01, and at the HLA-C locus, alleles 01:02, 03:02 and 12:02, were also found at statistically significantly different allele frequencies only when NPC cases were compared with EBV-negative controls. This suggests that these alleles, identified by GWAS as associated with development of NPC, may also be associated with EBV infection or humoral immunity [87].

### Gastric carcinoma

EBV is found in 10% of GC, a malignant tumour of the stomach epithelium [88]. EBV-positive GC is more prevalent in men than in women [73,89]. Both the EBV-positive and negative forms of GC have a marked geographical distribution, being highly prevalent in Japan and the Andes. High EBV antibody titres are seen in GC cases [88]. Given the high prevalence of EBV-positive GC [90], very little is known about whether differences exist in the genetic risk of developing EBV-positive versus EBV-negative GC and whether they have different epidemiological risk profiles [91]. Many genes have been found to be somatically mutated in EBV-positive GC [92], but these mutations were not found in non-tumour tissue samples. Promoter polymorphisms in *TNF* and *IL10* have been found to differ by EBV status in a candidate gene study of EBV-positive (30 patients) and EBV-negative GC (120 patients) and 220 healthy controls. The high producer *TNF* allele is associated with EBV-positive GC, whereas the *IL10* high producer allele is found more often in EBV-negative GC [93]. Future GWAS of GC risk may find that stratifying GC by EBV status reveals different genetic associations.

## Common Threads in Epstein–Barr Virus Infection and Immunity

**Figure 1:**
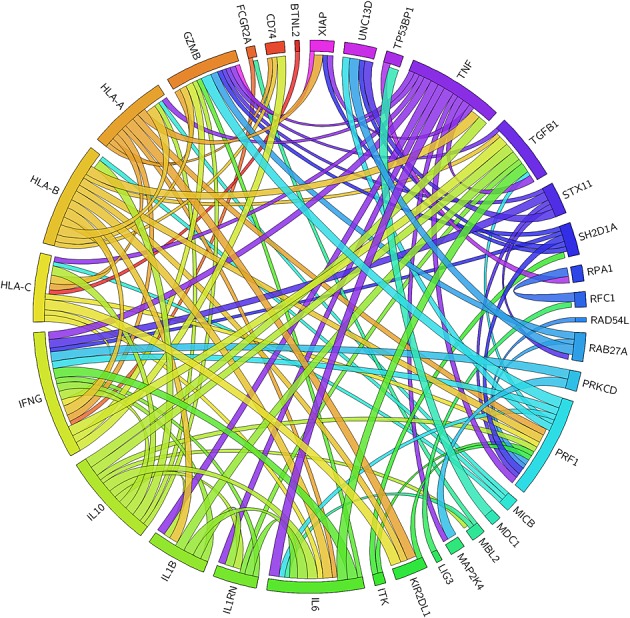
Circos plot [95] of protein–protein interactions of EBV-associated genes. Predicted protein–protein interactions (STRING [94]) of the EBV infection and disease-associated genes summarised in this paper. Many of the genes identified are found in the same interactome, which may indicate common pathways involved in the development of EBV-associated diseases

The role of host genetic variation in EBV-associated disease is still an emerging field of study. Many of the studies reviewed here draw their evidence from case reports of private mutations or from small cohorts from a single geographic area. They have typically employed a candidate gene approach, using either current knowledge of EBV biology or results from previous studies of EBV-related disease, to identify polymorphisms putatively involved in the response to EBV infection. They have nevertheless identified a number of interesting host gene variants associated with EBV disease (Table 2). The protein–protein interactions (predicted by Search Tool for the Retrieval of Interacting Genes/Proteins (STRING) [94]) between these genes are shown [95] in Figure1.

**Table 2:** Human genes linked to EBV-related disease

EBV-associated trait or disease	Gene	Reference
EBV antibodies	*BAT2*	Rubicz [28]
EBV antibodies	*BTNL2*	Rubicz [28]
Coronin-1A deficiency	*CORO1A*	Moshous [44]
Hypogammaglobulinaemia, NPC	*CR2*	Thiel [111], Fan [86]
EBV antibodies	*EHMT2*	Rubicz [28]
Low-grade B-cell lymphoma, EBV + Hodgkin lymphoma	*FCGR2A*	Diamantopoulos [80], Ghesquieres [78]
Haemophagocytic lymphohistiocytosis	*GZMB*	Zaitsu [56]
EBV + Hodgkin lymphoma	*HCG9*	Urayama [75]
Infectious mononucleosis, multiple sclerosis, EBV antibodies	HLA	McAulay [5],
EBV + Hodgkin lymphoma, post-transplant lymphoproliferative disorders, EBV + Hodgkin lymphoma, NPC	HLA-A	Niens [74], Reshef [65], Huang [112], Tang [87], Zivadinov [113]
Post-transplant lymphoproliferative disorders, EBV antibodies, NPC	HLA-B	Reshef [65], Durovic [29], Tang [87]
NPC, EBV antibodies	HLA-C	Tang [87]
EBV antibodies, post-transplant lymphoproliferative disorders, EBV + Hodgkin lymphoma, MS	HLA-DR	Nielsen [25], Rubicz [28], Hocker [67], Diepstra [35], De Jager [114]
PTLD	*IFNG*	Lee [72]
EBV antibodies, EBV viraemia	*IL1B*	Hurme [26], Kasztelewicz [68]
EBV viraemia, NPC	*IL1RN*	Kasztelewicz [68], Sousa [85]
EBV + Hodgkin lymphoma	*IL6*	Cozen [77]
Infectious mononucleosis, EBV antibodies, EBV + Hodgkin lymphoma, PTLD, GC	*IL10*	Yasui [24], Helminen [37], Babel [69], Silva [76], Wu [93]
IL-2-inducible T-cell kinase deficiency	*ITK*	Huck [17]
Haemophagocytic lymphohistiocytosis	*KIR2DS5*	Qiang [57]
EBV antibodies	*LIG3*	Shen [22]
CAEBV	*MAGT1*	Li [41]
Infectious mononucleosis, EBV antibodies	*MBL2*	Friborg [27]
EBV antibodies	*MDC1*	Shen [22]
NPC	*MAP2K4*	Zheng [83]
EBV + Hodgkin lymphoma	*MICB*	Urayama [75]
Chronic active EBV, haemophagocytic lymphohistiocytosis	*PRF1*	Katano [48],
Chronic active EBV	*PRKCD*	Kuehn [46]
Haemophagocytic lymphohistiocytosis	*RAB27A*	Szczawinska-Poplonyk [60]
EBV antibodies	*RAD54L*	Shen [22]
EBV antibodies	*RFC1*	Shen [22]
EBV antibodies	*RPA1*	Shen [22]
X-linked lymphoproliferative disorder	*SH2D1A*	Booth [38]
Haemophagocytic lymphohistiocytosis	*STX11*	Albayrak [58]
Infectious mononucleosis, PTLD	*TGFB1*	Hatta [36], Babel [69]
PTLD, gastric carcinoma, EBV-Hodgkin lymphoma survival	*TNF*	McAulay [71], Wu [93], Ghesquieres [115]
EBV antibodies	*TP53BP1*	Shen [22]
Haemophagocytic lymphohistiocytosis	*UNC13D*	Zhizhuo [59]
X-linked lymphoproliferative disorder	*XIAP*	Rigaud [40]

Some well-powered studies of susceptibility to EBV-associated cancers have begun to leverage the power of genome-wide approaches to understanding disease pathogenesis; by analysing EBV-positive and EBV-negative forms of different cancers as discrete groups, it is possible to begin to tease out the contribution that EBV makes to these conditions, for example, Huang *et al.* and Urayama *et al*. [75,96]. It is tempting to speculate in some cases that common pathways may influence different EBV diseases.

To date, there are more than 30 host genes that have been associated with EBV infection, immunity and disease. Those that are of potentially the greatest interest are the genes that have been associated with more than one EBV-related pathology or aspect of EBV immunity. Here we distil such genes and variants with disease overlap.

### Fc fragment of IgG, low affinity IIa, receptor associated with Hodgkin's and non-Hodgkin's lymphoma

*FCGR2A* is present on the cell surfaces of macrophages, neutrophils and NK cells, with roles in phagocytosis and modulation of the immune response. The studies linking this gene to EBV-positive B-cell lymphoma have both been relatively small and have examined a disparate collection of classical HL and non-HL, non-BL, making it difficult to draw conclusions on the role of *FCGR2A* in EBV susceptibility and disease [78,80]. SNP rs1801274 is a functional polymorphism affecting binding affinity of this IgG receptor, encoding a histidine to arginine substitution. The histidine-encoding allele is also associated with Kawasaki disease, an autoimmune disorder of unknown aetiology in which EBV may play a role [97,98].

### Human leukocyte antigen system and association with infectious mononucleosis, multiple sclerosis, Epstein–Barr virus antibodies, Hodgkin's lymphoma, post-transplant lymphoproliferative disorder and nasopharyngeal carcinoma

Large [28] and small [29] studies of EBV antibody responses have identified association with polymorphisms with the HLA system (including class I and II polymorphisms), which are so far independent from the polymorphisms identified as driving other EBV-related disease [28].

### Interleukin 10 and association with infectious mononucleosis, Epstein–Barr virus antibodies, Hodgkin's lymphoma, post-transplant lymphoproliferative disorder and gastric carcinoma

Given the broad range of functions that the anti-inflammatory cytokine IL10 fulfils in the human immune system, it is not surprising that polymorphisms in *IL10* have been popular candidates in EBV infection and disease susceptibility studies. However, the effect of different *IL10* genotypes on EBV disease is not clear. Minnicelli *et al.* found independent effects of *IL10* genotype and EBV status on BL outcomes [99]. A small study of organ transplant recipients did not find a significant association between IL10 expression and progression to chronic high EBV loads [100]. Other studies have found no association between *IL10* genotype and HL [101]. These studies share a common feature of small sample sizes, which may explain their conflicting results, as they are less robust to statistical errors if the effect in question is small or rare.

### Perforin and association with chronic active Epstein–Barr virus and haemophagocytic lymphohistiocytosis

*PRF1* permits granzymes A and B to reach the cytoplasm of cells targeted for destruction [102]. *PRF1* mutations are found in 30% of HLH cases and may be important to general protection from herpes virus-driven lymphoproliferation [54,103,104]. For example, Kaposi's sarcoma-associated herpesvirus-positive HLH occurred in two siblings with *PRF1* mutations [105]. The association with GZMB polymorphisms and HLH suggests an important role for targeted cell death in control of EBV infection.

### Tumour necrosis factor alpha and association with post-transplant lymphoproliferative disorder and gastric carcinoma

Small studies of two different conditions (PTLD, GC) have linked host SNPs within *TNF* (a major inflammatory cytokine) to variable susceptibility to these EBV-related disorders. *In vitro*, EBV immediate-early lytic gene BZLF1 down-regulates the TNFα receptor, preventing TNFα-induced cell death and signalling [106]. However, no GWAS of EBV-related conditions have reported an association with *TNF* polymorphisms.

## Conclusions

To further our understanding of the mechanisms by which EBV causes pathology and predict which individuals are at greatest risk of EBV-mediated disease, an increased focus on host genetics of EBV infection is required. The methodology of these studies must shift from candidate genes interrogated for associations with a disparate range of disorders in small populations to genome-wide approaches. Only large, well-powered studies can reliably identify common genetic polymorphisms contributing to the risk of EBV-related disease, with the hope of improving screening and treatment of these conditions. As the cost of such studies continues to fall, the next 50 years of EBV research may see more progress in this area.

## Abbreviations

BL: Burkitt's lymphoma
*BTNL2*: Butyrophilin-like 2 (MHC class II associated)
CAEBV: Chronic active Epstein–Barr virus
*CORO1A*: Coronin-1A
*CR2*: Complement component receptor 2
EBNA-1: Epstein–Barr nuclear antigen one
*EHMT2*: Euchromatic histone-lysine N-methyltransferase 2
*FCGR2A*: Fc fragment of IgG, low affinity IIa, receptor (CD32)
GC: Gastric carcinoma
GWAS: Genome-wide association study
GZMB: Granzyme B
HCST: Haematopoietic stem cell transplant
HL: Hodgkin's lymphoma
HLH: Haemophagocytic lymphohistiocytosis
HRRS: Homologous recombination repair system
*IL1B*: Interleukin 1 beta
*IL1RN*: Interleukin 1 receptor antagonist
*IL6*: Interleukin 6
*IL10*: Interleukin 10
IM: Infectious mononucleosis
*IFNG*: Interferon gamma
*ITK*: IL-2-inducible T-kinase
LCL: Lymphoblastoid cell line
*LIG3*: Ligase III, DNA, ATP-dependent
LMP1: Latent membrane protein 1
*MAGT1*: Magnesium transporter 1
*MAP2K4*: Mitogen-activated protein kinase kinase 4
*MBL2*: Mannose-binding lectin 2
*MDC1*: Mediator of DNA-damage checkpoint 1
MS: Multiple sclerosis
NPC: Nasopharyngeal carcinoma
*PRKCD*: Protein kinase C delta
*PRF1*: Perforin
PTLD: Post-transplant lymphoproliferative disorder
OR: Odds ratio
*RAB27A*: RAB27A-like
*RAD54L*: DNA repair and recombination protein RAD54-like
*RFC1*: Replication factor C (activator 1) 1
*RPA1*: Replication protein A1
SCID: Severe combined immune deficiency
*SH2D1A*: SH2 domain containing 1A
SNP: Single nucleotide polymorphism
STR: Short tandem repeat
*STX11*: Syntaxin 11
*TGFB1*: Transforming growth factor, beta 1
TLR: Toll-like receptor; TLR3 Toll-like receptor 3
*TP53BP1*: Tumour protein p53 binding protein 1
TRAF: TNF receptor associated factors
TRIF: TIR-domain-containing adapter-inducing interferon-β
*UNC13D*: Unc-13 homolog D
VCA: Viral capsid antigen
VNTR: Variable number tandem repeat
XMEN: X-linked magnesium deficiency with EBV infection and neoplasia
*XIAP*: X-linked inhibitor of apoptosis
XLP: X-linked lymphoproliferative syndrome
